# Cortistatin hyperpolarizes pancreatic beta cell membrane and reduces glucose-stimulated insulin secretion

**DOI:** 10.1186/1758-5996-7-S1-A250

**Published:** 2015-11-11

**Authors:** Alex Rafacho, Manuel Castellano-Muñoz, Paloma Alonso-Magdalena, Esperanza Irles, Melisa Bello, Jean Vetorazzi, Beatriz Merino, Sergi Soriano, Pau Iborra, Antonia Ruiz, Raúl M Luque, Angel Nadal, Ivan Quesada

**Affiliations:** 1 Universidade Federal de Santa Catarina, Florianópolis, Brazil

## Background

Cortistatin-14 (CORT) is a neuropeptide commonly expressed in inhibitory neurons of the central nervous system (CNS) with structural, pharmacological and functional similarity to somatostatin (SST). In addition to having roles in the CNS, both peptides also regulate endocrine secretion. Yet the cellular mechanisms supporting this role are not well understood.

## Objectives

We studied the potential role of CORT in pancreatic beta and alpha cells function.

## Materials and methods

Isolated islets and primary pancreatic beta cells from lean C57BL6 mice were used for determination of functional and electrophysiological parameters.

## Results

Using insulin and glucagon secretion protocols with fresh islets isolated from C57BL6 mice, we observed that CORT reduced the glucose-stimulated insulin secretion (GSIS) in a similar magnitude from that of SST (p<0.01), an effect mediated by SST-R5 receptor. Glucagon secretion in response to 0.5 mM glucose was completely abrogated in the presence of CORT (p<0.001), as well as for SST. Beta cell function were further investigated and we observed that the reduction in insulin secretion was paralleled by a decrease in the glucose-induced calcium levels observed by fura-2 calcium imaging (p<0.001). As opposed to the effects on SST, the effect of CORT in beta cell calcium load was blocked by specific SST-R5 receptor antagonist, suggesting a higher affinity of CORT for this receptor. In addition, CORT reduced beta cell membrane potential and abolished action potential firings in perforated patch clamp experiments (p<0.001). CORT also diminished calcium currents in whole cell patch clamp experiments.

## Conclusion

Our results suggest that the binding of CORT to SST-R5 receptors Results in beta cell hyperpolarization and impairs calcium channels activity, thus reducing the beta-cell stimulus secretion coupling.

